# The Role of Glycocalyx and Caveolae in Vascular Homeostasis and Diseases

**DOI:** 10.3389/fphys.2020.620840

**Published:** 2021-01-13

**Authors:** Simone Regina Potje, Tiago Dal-Cin Paula, Michele Paulo, Lusiane Maria Bendhack

**Affiliations:** Faculty of Pharmaceutical Sciences of Ribeirão Preto, University of São Paulo, Ribeirão Preto, Brazil

**Keywords:** glycocalyx, caveolae, vascular tone, ROS, hypertension, atherosclerosis, endothelial cells, eNOS

## Abstract

This review highlights recent findings about the role that endothelial glycocalyx and caveolae play in vascular homeostasis. We describe the structure, synthesis, and function of glycocalyx and caveolae in vascular cells under physiological and pathophysiological conditions. Special focus will be given in glycocalyx and caveolae that are associated with impaired production of nitric oxide (NO) and generation of reactive oxygen species (ROS). Such alterations could contribute to the development of cardiovascular diseases, such as atherosclerosis, and hypertension.

## Introduction

The role that endothelium plays in modulating the vascular tone includes the synthesis and release of several vasoactive substances, especially the vasodilator nitric oxide (NO) (Cahill and Redmond, 2016). Endothelial NO synthase (eNOS) is responsible for the synthesis of most of the NO that is produced in endothelial cells (ECs) (Zhao et al., 2015). eNOS is localized on domains named caveolae, which are spread over the entire ECs surface (Shaul, 2003). The glycocalyx is a polysaccharide-rich layer, which underlies mechano-transduction and mediates the physiological activation of NO synthesis by shear stress (Pahakis et al., 2007). More specifically, the glycocalyx components transform mechanical signals into biochemical signals, to activate eNOS (Florian et al., 2003; Pahakis et al., 2007), thereby contributing to vascular homeostasis (Alphonsus and Rodseth, 2014).

Shedding of glycocalyx and changes in the structure of caveolae decreases eNOS activity, which reduces NO bioavailability and generates reactive oxygen species (ROS) (Kumagai et al., 2009; Potje et al., 2019). Both consequences are associated with cardiovascular diseases such as atherosclerosis and hypertension. Therefore, the organization and function of glycocalyx and caveolae might be altered in atherosclerosis and hypertension, which results in release of deleterious ROS that contribute to these pathological conditions.

This review aims to highlight recent findings about the activation of glycocalyx and caveolar enzymes that participate in the synthesis and release of NO and ROS and alterations that could impair the proper function of glycocalyx and caveolae in pathological conditions like atherosclerosis and hypertension.

## Structure and Synthesis of Endothelial Glycocalyx

As reviewed by different groups, the endothelial glycocalyx is mainly composed of glycosaminoglycans, proteoglycans, and glycoproteins. Heparan sulfate, chondroitin sulfate, and hyaluronic acid chains constitute glycosaminoglycans. Proteoglycans include core protein families such as perlecan, syndecans-1, -2, -3, -4, and glypican-1. Lastly, glycoproteins consist of sialic acid oligosaccharides (Uchimido et al., 2019; Möckl, 2020).

Heparan sulfate is the predominant constituent (from 50 to 90%) of glycocalyx (Reitsma et al., 2007). The syndecan family can contain three to eight potential heparan sulfate or chondroitin sulfate attachment sites depending on the specific syndecan member. These sites are located close to the syndecan NH_2_-terminal ectodomain or adjacent to the transmembrane domain near the syndecan COOH terminal. Glypican-1 is the only proteoglycan that is expressed exclusively in ECs. It binds specifically to the heparan sulfate chain and is localized in lipid rafts (caveolae) through a C-terminal glycosylphosphatidylinositol (GPI) anchor (Uchimido et al., 2019; Möckl, 2020).

Biosynthesis of glycosaminoglycans is a complex process that is initiated by chain polymerization and which depends on various stepwise reactions like sulfation and epimerization. This process happens in many cellular components including endoplasmic reticulum and Golgi apparatus, which are responsible for the secretory pathway (Uchimido et al., 2019; Möckl, 2020). On the other hand, hyaluronic acid is directly assembled in the membrane by hyaluronan synthases, and it is secreted into the extracellular space (Agarwal et al., 2019).

## Vascular Protective Effects of Glycocalyx

Glycocalyx functions as a vascular protector because it participates in angiogenesis, exerts an anticoagulant effect, prevents leukocyte adhesion, acts as a selective permeable barrier and filter, operates as a mechano-transducer of shear stress, and contributes to maintaining the vascular tone.

Glycocalyx, specifically heparan sulfate, regulates angiogenesis by playing a proangiogenic role (Fuster and Wang, 2010). 6-O-sulfation of heparan sulfate is an essential regulator of vascular morphogenesis in zebrafish (Chen et al., 2005). In addition, decreased heparan sulfate N-sulfation impairs recruitment of pericytes and development of vasculature in N-deacetylase/N-sulfotransferase (Ndst)-1 knockout mice (Abramsson et al., 2007). Moreover, complete loss of heparan sulfate chains in mural cells causes embryonic death in the late stages of vascular morphogenesis and stability (Stenzel et al., 2009). In this way, glycocalyx contributes to angiogenesis process.

Antithrombin III is the main anticoagulant molecule that can bind to specific sites of heparan sulfate; it also inhibits coagulant factors and inactivates factors IX and X (Shimada et al., 1991; Quinsey et al., 2004). Likewise, tissue factor pathway inhibitor (TFPI) can also bind to heparan sulfates and block the initial steps of blood coagulation by inhibiting factors VIIa and Xa (Kato, 2002). Additionally, dermathan sulfate in glycocalyx can activate heparin cofactor II, which inhibits thrombin (Tovar et al., 2005). Furthermore, degradation of endothelial glycocalyx induced by hyperglycemia activates coagulation in healthy subjects (Nieuwdorp et al., 2006b). Therefore, glycocalyx has anticoagulant and antithrombotic effects.

The glycocalyx layer has consistency and anti-adhesive character, promoting resistance to penetration of circulating leukocytes and preventing leukocyte-endothelial adhesion in vascular smooth muscle cells (VSMCs). Besides, degradation of the glycocalyx layer provoked by heparitinase in mouse cremaster venules increases leukocyte adhesion in a dose-dependent manner (Constantinescu et al., 2003). In addition, enzymatic degradation of glycocalyx promoted increased of ICAM-1 expression, which was associated to a de-regulation in NF-κB activity in response to flow and leukocyte adhesion (McDonald et al., 2016). Moreover, endotoxemia stimulated in mice by tumor necrosis factor-α (TNF-α) rapidly degrades pulmonary microvascular glycocalyx, which contributes to neutrophil adhesion (Schmidt et al., 2012). Consequently, the damage of glycocalyx favors the adherence of leukocyte on the ECs.

Tumor necrosis factor-α treatment increases porosity and permeation due to glycocalyx shedding with enhanced intraluminal volume (Henry and Duling, 2000). Patients with type 1 diabetes show glycocalyx damage in sublingual capillaries, which is associated with microalbuminuria (Nieuwdorp et al., 2006a). Furthermore, degradation of heparan sulfate by heparanase promotes injury in porcine aortic ECs, which was associated to apoptosis and cell death (Han et al., 2005). Thus, glycocalyx works as a barrier and filter, besides protecting vascular cells.

Shear stress on ECs is a frictional force (mechanical signals) per unit area created by laminar blood flow (Pohl et al., 1986). Heparan sulfate is important to detect the direction of shear stress because degradation of this substance prevents shear stress-induced directional migration of ECs and inhibits recruitment of phosphorylated focal adhesion kinase in the flow direction (Moon et al., 2005). Nevertheless, remodeling of glycocalyx in response to short and long periods of shear stress has been reported (Liu et al., 2016). Moreover, reorganization of actin cytoskeleton and focal adhesions in response to fluid shear stress has been shown in rat fat-pad ECs in various flow media (Thi et al., 2004). Similarly, changes in the actin cytoskeleton and caveolae have been demonstrated after long-term shear stress (24 h), which also redistributes and restores heparan sulfate, syndecan-1, and glypican-1 on the apical surface of ECs (Zeng and Tarbell, 2014). In this way, the actin cytoskeleton contributes to the structural stability of glycocalyx under shear stress (Li and Wang, 2018).

Mechano-transduction is the conversion of mechanical signals induced by shear stress into biochemical signals inside ECs (Dabagh et al., 2017). Endothelial glycocalyx has been described as the primary sensor activating the mechano-transduction process, creating an immediate response to shear stress stimulus and producing NO. Removing heparan sulfate and other glycocalyx components through a selective enzyme that degrades endothelial glycocalyx constituents blocks shear-induced NO production in ECs (Florian et al., 2003; Pahakis et al., 2007; Yen et al., 2015; Dragovich et al., 2016). Furthermore, NO production mediated by glycocalyx is associated with calcium influx mediated by endothelial transient receptor potential (TRP) channels. Under stimulation, the proteoglycans promote tension in the lipid bilayer, which spreads through ECs due to its interaction with cytoskeleton, and then both proteoglycans and cytoskeleton may activate a diversity of mechanically sensitive ion channels, such as TRP channels (Dragovich et al., 2016). In addition, reduced NO production induced by flow has been reported in isolated canine femoral arteries treated with hyaluronidase, which degrades the hyaluronic acid GAG (Mochizuki et al., 2003). These results show that intact glycocalyx, mainly heparan sulfate chains, are needed to activate eNOS and thus produce NO.

Glypican-1 seems to be the main heparan sulfate proteoglycan that is associated with NO production, along with eNOS, both resides in caveolae. First, glypican-1 knockdown blocks eNOS activation under shear stress stimulus (Ebong et al., 2014). Additionally, glypican-1 removal significantly suppresses eNOS activation mediated by several steady shear stress magnitudes (Zeng and Liu, 2016). Besides that, atomic force microscopy (AFM) selectively applied on glypican-1 for a limited time significantly increases NO production, whereas pulling on syndecan-1, CD44, and hyaluronic acid does not change NO concentration (Bartosch et al., 2017). Furthermore, disturbed flow (DF) reduces caveolin-1 (Cav-1) expression and impairs its co-localization with eNOS, consequently reducing eNOS phosphorylation at Serine^1177^ (Harding et al., 2018). Taken together, these results indicate that glypican-1 is a primary mechano-sensor for shear stress-induced NO production, and that the glypican-1-caveolae-eNOS-NO pathway is essential for vascular tone maintenance.

## Formation of Caveolae

Lipid rafts (also known as lipid microdomains) and caveolae are domains of the plasma membrane that share the same composition, such as cholesterol, sphingolipids, and glycosyl-phosphatidylinositol GPI-anchored proteins. However, the caveolae structure is an invagination at the membrane. On the other hand, lipids rafts are flat areas of the membrane (Bieberich, 2018). Caveolae were first described in the 1950s by using an electron microscope. Due to lack of experimental approaches and technologies, the caveolar functions remained mostly unclear until the 1990s (Anderson, 1998). Now, caveolae are defined as 60–80-nm-wide pits in the plasma membrane that contain oligomeric caveolin (Parton and Simons, 2007). Caveolae are predominantly expressed in vascular ECs, but they are also present in VSMCs (Gratton et al., 2004). Molecular understanding of caveolar formation is advancing rapidly, and we now know that sculpting the membrane to generate the characteristic bulb-shaped caveolar pit involves coordinated action of integral membrane proteins and peripheral membrane coat proteins in a process that depends on their multiple interactions with membrane lipids (Parton et al., 2018).

Three mammalian caveolins exist: Cav-1, Cav-2, and Cav-3. Cav-1 and Cav-2 are generally expressed together in different types of cells other than muscle cells, whereas Cav-3 is predominantly expressed in muscle cells (Razani and Lisanti, 2001). Some cells, including smooth muscle cells and cardiomyocytes, can express Cav-1, Cav-2, and Cav-3, (Head et al., 2006; Robenek et al., 2008). Each caveola has estimated 140–150 Cav-1 molecules (Pelkmans and Zerial, 2005). Cav-1 loss results in complete absence of caveolae (Drab et al., 2001). Moreover, Cav-1 expression in cells without caveolae causes caveolae to form (Vogel et al., 2019). Therefore, Cav-1 is crucial for caveolar formation.

Identifying the family of cytoplasmic proteins that cooperatively work with caveolins for caveolar formation and function has expanded our understanding of caveolar biology. Liu et al. (2008) described that Cavins are cytoplasmic proteins with amino-terminal coiled-coil domains that play a role as protein component of caveolae, where they form large heteromeric complexes that are recruited by caveolins in cells expressing caveolae (Bastiani et al., 2009). The Cavin family includes Cavin-1 (also known as polymerase I and transcript release factor, PTRF), Cavin-2 (SDPR, serum deprivation response protein), Cavin-3 (also known as related gene product that binds to c-kinase - SRBC), and Cavin-4 (also known as muscle-restricted coiled-coil protein, MURC). Cavin knockout mice are viable, but they present a lipodystrophic phenotype with high triglyceride levels, glucose intolerance, and hyperinsulinemia (Liu et al., 2008). In addition, caveolae are completely absent in Cavin knockout mice in specific tissues like lung epithelium, intestinal smooth muscle, skeletal muscle, and ECs. In this way, formation of caveolae requires Cavin-1 (Liu et al., 2008). Cavin-2 and Cavin-3 have been identified as protein kinase C (PKC) substrates and have been suggested to target PKC for caveolae. Cavin-2 has been associated with caveolar membrane curvature, and Cavin-3 affects formation of caveolar endocytic carriers (Hill et al., 2008). Cavin-4, which is predominantly expressed in cardiac and skeletal muscles, has been related to myogenesis and muscle hypertrophy via RHOA–RHO-associated kinase (ROCK), ERK1, and ERK2, as well as to regulation of atrial natriuretic peptide transcription in cardiac muscle (Ogata et al., 2008; Bastiani et al., 2009).

After trafficking to the plasma membrane, caveolin oligomers are stabilized by the complex of Cavins (Hayer et al., 2010). Lipids and/or membrane lipid order may also be important for this interaction. The four members of the Cavin family bind to phosphatidylserine, which is abundant on the cytoplasmic face of the plasma membrane, particularly in areas that are rich in caveolae (Fairn et al., 2011). Cav-1 peptides can generate phosphatidylserine domains in liposomes, so membrane lipid reorganization by caveolins might also contribute to a stable interaction in the plasma membrane (Wanaski et al., 2003). In this way, Cavins and caveolins preserve the stable coat around the bulb of caveolae (Hill et al., 2008). Additionally, a protein called Eps15 homology domain protein 2 (EHD_2_) is involved in mediating caveolar stabilization in the plasma membrane (Stoeber et al., 2012). Moreover, the protein pacsin2 participates in membrane bending to form caveolae and to release NO from eNOS-expressing cells (Hansen et al., 2011), thus it reduces vascular tone *ex vivo* and lowers blood pressure in mice (Bernatchez et al., 2011).

The way through which caveolae suffer endocytosis has been a subject of controversy for many years. However, a consensus has emerged that dynamin drives caveolar budding from the surface. In ECs, caveolae have been proposed to bud in from the luminal surface and to fuse with the abluminal surface, to mediate efficient trans-endothelial transport from the blood stream to the underlying tissues. In other cell types, caveolae fuse with early endosomes (Rippe et al., 2002; Oh et al., 2007).

## Caveolae and Signal Transduction Molecules

Recently, the structures of caveolae and caveolin proteins have been discovered to play an important role in cellular physiological functions, particularly functions related to cholesterol transport, endocytosis, tumor suppression, and cell signal transduction (Lian et al., 2019).

Signal transduction is promoted by neurotransmitters, circulating hormones, and growth factors that are critical for the regulation of vasculature. Many such regulators act by interacting with plasma membrane receptors and subsequently perturbation pathways that modulate metabolic activity, growth, death, and differentiated functions of the target cells (Insel and Patel, 2009).

Membrane rafts and caveolae concentrate a subset of membrane constituents, including proteins and other components involved in transport and signal transduction (Allen et al., 2007; Patel et al., 2008). Lipid rafts have non-homogeneously organized signaling, which facilitates temporally and spatially efficient cellular regulation by extracellular hormones and growth factors. The interior of cells has gradients of second messengers and effectors (cyclic AMP, Ca2þ, protein kinases/phosphatases, and phosphodiesterases) that participate in vascular signaling (Bauman et al., 2006; Echarri et al., 2007). Membrane rafts that lack caveolins also concentrate signaling molecules, implying that other factors (e.g., binding to lipids) contribute to the interaction of signaling entities with rafts and caveolins (Patel et al., 2008).

In several types of cells, including ECs and VSMCs, mediators of Ca^2+^ signaling such as Ca^2+^-ATPase, inositol 1,4,5-trisphosphate receptors, Ca^2+^ pumps, L-type Ca^2+^ channels, large-conductance Ca^2+^-activated K^+^ channels, calmodulin, and TRP channels are localized in cholesterol-rich membrane domains (Wang et al., 2005). Moreover, in VSMCs, caveolae are closely associated with peripheral sarcoplasmic reticulum, a major site for Ca^2+^ release that has been postulated to be the preferred site of Ca^2+^ entry in response to Ca^2+^ depletion (Shaw et al., 2006). These observations suggest that membrane rafts and caveolae have a role in Ca^2+^ signaling.

According to Durr et al. (2004), various proteins like G-protein-coupled receptor (GPCR) and downstream signaling enzymes such as eNOS are specifically enriched in caveolae in ECs. Additionally, caveolae contribute to GPCR desensitization and internalization (Chini and Parenti, 2004). For example, the stimulation with angiotensin II (Ang II) promotes rapid translocation of AT_1_ receptor (AT_1_R) to caveolae, then AT_1_R bind to Cav-1, which delays AT_1_R reactivation after prolonged stimulus with Ang II (Ishizaka et al., 1998; Czikora et al., 2015).

## Contribution of eNOS and Cav-1 to NO Generation

Controlling eNOS activation falls under a complex regulatory mechanism that includes tonic inhibitory interaction with Cav-1 (Ju et al., 1997) and post-translational modifications like myristoylation, palmitoylation, phosphorylation, and stimulatory responses, to raise intracellular Ca^2+^ concentrations (Sessa, 2004).

Endothelial NO synthase remains associated with Cav-1, which is the major component of caveolae. eNOS requires palmitoylation and myristoylation to be targeted to the caveolar microdomains. The interactions between Cav-1 and eNOS have been shown to regulate NO release negatively (Grayson et al., 2012). In this way, Cav-1 over-expression decreases basal NO production in a “control” cellular state. Moreover, under agonist activation, eNOS translocates away from caveolae, thereby removing tonic Cav-1 inhibition (Frank et al., 2003). Feron et al. (1998) identified that, after agonist-dependent eNOS activation, removal of tonic inhibition between eNOS and Cav-1 coincides with de-palmitoylation concomitant with eNOS translocation to the non-caveolar fraction, which indicates increased NO biosynthesis. Conversely, when eNOS returns to the membrane/caveolae, it is re-palmitoylated, and its inhibitory interaction with Cav-1/eNOS is reasserted.

A model for activation of eNOS bound to Cav-1 considers that, under stimulation with Ca^2+^-mobilizing agonists, the inhibitory scaffold of Cav-1 is relieved via calcium-regulated binding of calmodulin and Hsp90 to displace eNOS from Cav-1, thus allowing efficient NO production (Sessa, 2004). Evidence supporting the inhibition model includes enhanced NO-dependent vascular function in blood vessels from Cav-1 knockout mice and increased NO production in ECs isolated from Cav-1 knockout mice, an effect that is rescued by Cav-1 reintroduction (Drab et al., 2001; Razani et al., 2001; Murata et al., 2007). Besides, transduction of cells or blood vessels with Cavtratin, a synthetic cell permeable Cav-1 CSD peptide, reduces NO release and inflammation *in vivo* (Bucci et al., 2006). Alanine scanning of this scaffolding region demonstrated that the threonine residues 90 and 91 (T90, T91) and phenylalanine 92 (F92) underlie eNOS inhibition. This is supported by lack of eNOS inhibition by the F92A–Cav-1 mutant in reconstituted cells and a Cavtratin-derived peptide with the T90/91 and F92 substitutions (a peptide called Cavnoxin) as revealed by studies *in vitro* and *in vivo* (Bernatchez et al., 2005).

Sowa (2012) showed that Cav-1 in caveolae but not in lipid rafts can inhibit eNOS under basal conditions. Although Cav-1 in caveolae keeps eNOS inactive, the specific localization of Cav-1 in this cell organelle is necessary for its activation (Chen et al., 2012). In addition, Cav-1/eNOS interaction is necessary to prevent inadequate NO production under basal conditions and to facilitate integration of extracellular stimuli with intracellular NO signals (Rath et al., 2009).

## Oxidative Stress in Cardiovascular Diseases

Reactive oxygen species are a group of heterogeneous molecules that are characterized by highly reactive oxygen atoms, short half-life, and strong capacity to engage in oxidation reactions (Vara and Pula, 2014). They are essential for homeostasis of the cardiovascular system and play a role in signaling pathways in different cells. An imbalance in antioxidant and oxidant systems promotes ROS overproduction, which culminates in oxidative stress, a well-known and important hallmark of cardiovascular diseases (Panth et al., 2016). When ROS levels overtake the cellular defenses, protein, lipids, and DNA can undergo oxidation, which can lead to cellular damage, tissue injury, and inflammation (Sena et al., 2018). ROS are produced by distinct enzymatic sources like xantine oxidase, NADPH-oxidase (NOX), cyclooxygenase (COX), lipoxygenase (LOX), monomeric eNOS (uncoupled eNOS), myeloperoxidase, and also by the respiratory chain in mitochondria (Vara and Pula, 2014; Sena et al., 2018). The chemical species anion superoxide (O_2_^–^), peroxynitrite (ONOO^–^), hydrogen peroxide (H_2_O_2_), and hydroxyl radical (•OH) underlie deleterious effects of oxidative stress. However, ROS present not only deleterious, but also physiological effects on vascular tone in VSMCs and on ECs motility, proliferation, and permeability (Vara and Pula, 2014). For example, O_2_^–^ induces protein kinase-dependent contraction in VSMCs under high pressure (Ungvari et al., 2003), whereas H_2_O_2_ upregulates vascular endothelial growth factor receptor 2 (González-Pacheco et al., 2006).

Each oxidant chemical species can be removed from the cellular environment by different enzymes that make up the antioxidant system, including superoxide dismutase (SOD), catalase (CAT), glutathione peroxidase, thioredoxin, peroxiredoxin, and glutathione transferase. SOD dismutates O_2_^–^ in H_2_O_2_, which is broken down into O_2_ and H_2_O by CAT and glutathione peroxidase (Birben et al., 2012). The radical species can also activate the nuclear factor erythroid 2-like 2 (Nrf-2), a transcription factor that is involved in the dynamic regulation of the antioxidant system, thereby activating the expression of promoters containing the antioxidant response element (Satta et al., 2017).

In the vascular system, both ECs and VSMCs can be either producers or targets of ROS. H_2_O_2_ is produced by NOX4 in ECs (Burtenshaw et al., 2019) and it can elicit different responses in VSMCs depending on its concentration (Gil-Longo and González-Vázquez, 2005). Importantly, H_2_O_2_ seems to be increased in aortas from hypertensive rats under stimulus (Silva et al., 2013). O_2_^–^ can be produced by NOX isoforms expressed in the membrane and in the intracellular compartment of ECs (Li and Shah, 2002) and by mitochondria, which are considered the major source of O_2_^–^ in ECs (Li et al., 2016). In cardiovascular diseases, O_2_^–^ significantly contributes to endothelial dysfunction because it rapidly reacts with NO, to produce the highly oxidizing ONOO^–^ (Radi, 2018), thus decreasing NO bioavailability. In addition, ROS can induce conversion of ECs to myofibroblasts, losing its endothelial properties (Montorfano et al., 2014).

Endothelial dysfunction is characterized by reduced endothelial response to different stimulus that release NO and other chemical mediators related to vasodilation or higher levels of endothelial chemical mediators associated with vasoconstriction (Vanhoutte et al., 2017). Therefore, exacerbated oxidative stress in ECs modifies the response of endothelial NO, and ROS produced by ECs can induce response in VSMCs.

In VSMCs, ROS production is mediated especially by NOX1 and NOX4 and produces O_2_^–^ and H_2_O_2_, respectively (Burtenshaw et al., 2019). Increased ROS in VSMCs is a common feature of different models of hypertension such as AngII-infused model (Rajagopalan et al., 1996;Zhou et al., 2020), spontaneously hypertensive rats (SHR) (Graton et al., 2019), renovascular hypertension (2K-1C) (Castro et al., 2012;Oliveira-Paula et al., 2016), and Doca-Salt rats (Amaral et al., 2015). Physiologically, contraction mediated by activation of α-1 adrenoceptors can partially depend on O_2_^–^ (Tsai and Jiang, 2010). The hypercontractile profile of VSMCs of hypertensive rats seems to depend on ROS production (Camargo et al., 2018). Additionally, ROS produced by VSMCs can reduce NO bioavailability.

## Oxidative Stress in Caveolae and Caveolin

Cav-1 seems to be involved in the process involving ROS as target or controlling ROS production. Oxidative stress mediated by H_2_O_2_ degrades Cav-1 in skeletal muscle cells (Mougeolle et al., 2015). H_2_O_2_ is increased in aorta of renal hypertensive rats (Silva et al., 2013), and the total number of caveolae is reduced in aorta of hypertensive rats (Rodrigues et al., 2010; Potje et al., 2019). Thus, H_2_O_2_ overproduction can be important to reduce Cav-1 levels and to disrupt the function of caveolae in hypertension.

Cav-1 is related to the AngII (AT1) receptor because the AT1-R⋅caveolin complex requires an intact caveolin scaffolding domain, but not co-localization in the caveolae (Wyse et al., 2003). Exposure to the agonist Ang II changes the Cav-1 levels in VSMCs (Ishizaka et al., 1998). Interestingly, Cav-1 loss increases NOX activity and ROS production in VSMCs (Zuo et al., 2005; Chen et al., 2014). In contrast, Cav-1 deletion can prevent remodeling induced by Ang II (Forrester et al., 2017). As stated before, Nrf-2 is an important element that controls the levels of antioxidant enzymes. ROS can activate Nrf-2 migration to the nucleus, thereby raising the expression of antioxidant enzymes and leading to detoxification of the cells (Satta et al., 2017). Curiously, Cav-1 seems to repress this migration given that Cav-1 knockout mice constantly present high Nrf-2 levels in nucleus (Volonte et al., 2013). Changes in Nrf-2 levels can alter the physiology of normal cells due to upstream and downstream of molecules that are associated with a defective Nrf2 signaling system (Satta et al., 2017). Thus, most of the common features of hypertensive vessels, e.g., endothelial dysfunction and hypercontractile VSMCs, can result from ROS actions, Cav-1 levels, and caveolar functions.

## Effect of ROS on Endothelial Glycocalyx Degradation

Specific enzymes, named sheddases, as well as metalloproteinases (MMPs), heparanase, and hyaluronidase degrade glycocalyx. Sheddases are activated by pro-inflammatory cytokines such as TNFα (Ramnath et al., 2014), interleukin-1beta (Haywood-Watson et al., 2011), interleukin-6, and interleukin-8 and also by shear stress, hypoxia, and ROS (Lipowsky and Lescanic, 2013). In this review, we focus only on how ROS affect endothelial glycocalyx given that ROS cleave and destabilize the glycocalyx structure (Sieve et al., 2018).

MMPs modify the constituents of glycocalyx, thus disrupting glycocalyx in pathological conditions (Lipowsky, 2011). MMPs can cleave the protein core of syndecan, promoting shedding of the syndecan family and consequent thrombosis, destabilization of vascular walls, endothelial dysfunction, and inflammation (Fitzgerald et al., 2000; Chen et al., 2017). In, addition, MMP-2 was associated to direct chondroitin sulfate cleavage (Hsu et al., 2006), while MMP-7 was responsible for cleavage of perlecan and heparan sulfate proteoglycans (Grindel et al., 2014) and MMP-9 mediated disruption of syndecan-4 (Ramnath et al., 2014). ROS also decrease the levels of the tissue inhibitors of metalloproteinases (TIMPs), thereby increasing the activity of MMPs (Siwik and Colucci, 2004). The inhibition of MMPs was able to restore the shedding of syndecan-4 in early diabetic disease (Ramnath et al., 2020).

Heparanase is an endoglycosidase that cleaves the side chains of heparan sulfate present in the syndecan and glypican families through hydrolysis, to disrupt glycocalyx (Garsen et al., 2014). Degradation of heparan sulfate reduces extracellular SOD (ecSOD), which remains attached to the heparan sulfate portion. ecSOD protects vascular cells from oxidative stress and its overexpression can attenuate heparanase expression, which suggests a prophylactic effect to prevent glycocalyx degradation (Kumagai et al., 2009).

Hyaluronidase degrades hyaluronic acid into fragments via hydrolysis of the disaccharides at hexosaminidic β (1–4) linkages (Wang et al., 2020). In addition, hyaluronic acid degradation products can produce ROS, which triggers several vascular disease processes (Soltés et al., 2006). Moreover, other ROS derived from O_2_^–^ and nitrogen monoxide (⋅NO), including H_2_O_2_, ONOO^–^, and hypochlorous acid, depolymerize hyaluronic acid (Uchiyama et al., 1990).

## Alterations in Caveolar Function and Glycocalyx in Atherosclerosis and Hypertension

Atherosclerosis and hypertension are multifactorial diseases. Atherosclerosis and its consequences represent the major cause of cardiovascular mortality. This disease is characterized by endothelial dysfunction, increased platelet adhesion, leukocyte recruitment, and accumulation of lipoproteins that evade phagocytosis (Eckardt et al., 2019). Endothelial dysfunction in the areas where atherosclerosis develops occurs through entry of lipoproteins, which is followed by lesions, leading to production of proinflammatory cytokines, migration of monocytes, and accumulation of macrophages (Gimbrone and Garcia-Cardeña, 2016). In turn, hypertension is a multifactorial disease that is associated with endothelial dysfunction, exacerbated oxidative stress, and inflammation in blood vessels (Dharmashankar and Widlansky, 2010). ECs play a pivotal role in vessel balance and pathophysiological conditions because they are exposed to inflammatory mediators that can impair or even destroy the endothelial layer and its components.

Caveolae and Cav-1 seem to play a part in atherosclerosis development. Numerous atherogenic proteins colocalize with caveolae in ECs, and caveolae are involved with transcytosis of low-density lipoprotein (LDL) particles (Frank et al., 2004; Sowa, 2012). Additionally, Cav-1 protein seems to have an atherosclerotic role. First, Cav-1 overexpression in ECs can increase atherosclerosis progression in apolipoprotein E-deficient mice (Fernández-Hernando et al., 2010). Moreover, the absence of Cav-1 promotes atheroprotection in vessels of Cav-1 knockout mice (Zhang et al., 2020). As proposed by Zhang et al. (2020), activation of endothelial autophagy by Cav-1 deficiency protects against atherosclerosis progression. In brief, autophagy is described as an evolutionarily conserved subcellular process that mediates degradation of proteins and damaged organelles via lysosomes (Mizushima and Komatsu, 2011).

As reported by Milovanova et al. (2008), apart from modulating NO production by eNOS, Cav-1 can modulate ROS production by NOX. In pulmonary hypertension, Cav-1 is a negative regulator of ROS derived from NO since lack of Cav-1 expression in pulmonary hypertension increases NOX activity and enhances ROS production (Chen et al., 2014). Furthermore, Cav-1 deletion prevents transactivation of hypertensive vascular remodeling and contributes to increased mitochondrial ROS levels in a model of AngII-induced hypertension (Forrester et al., 2017). On the other hand, lipid rafts and caveolae structural disruption with cholesterol disassembly drugs, increased ROS production in a different way than NOX. Recently, we have shown that caveolar structural disruption with methyl-β-cyclodextrin uncouples eNOS and raises ROS levels in Wistar normotensive rats and SHR aortas and mesenteric arteries (Potje et al., 2019). Besides that, the number of caveolae is reduced in renal hypertensive (2K-1C) and SHR rats as compared to normotensive rats, which impairs acetylcholine-induced endothelium-dependent relaxation and NO production (Rodrigues et al., 2010; Potje et al., 2019). The smaller number of caveolae could account for impaired NO donor-induced relaxation in 2K-1C rat aortas as compared to normotensive rat aortas (Rodrigues et al., 2007).

The literature contains controversial data about the role that Cav-1 has in the determination of arterial pressure. Whereas several authors do not report increased arterial pressure in Cav-1 knockout mice as compared to control wild-type (WT) mice, other authors describe lower arterial pressure in Cav-1 knockout mice than in WT mice (for a review, see Rahman and Sward, 2009). Cav-1 knockout mice present increased circulating NO levels and vasodilation, but the arterial pressure values measured by telemetry in awake mice and WT mice are similar (Desjardins et al., 2008). As proposed by Insel and Patel (2007), chronic Cav-1 deficiency could be compensated by other vascular mechanisms, to maintain the arterial pressure. Also, eNOS could be uncoupled in hypertensive vessels.

In physiological conditions, arterial ECs submitted to uniform flow (UF) release NO constantly (Noris et al., 1995). As described by Eckardt et al. (2019), the pathogenesis of atherosclerosis is associated with alterations in vascular glycocalyx. Glycocalyx degradation stimulates lipid flux, increasing lipid deposition in the arterial walls. This is associated with reduced eNOS expression, which decreases NO production and impairs vasodilation (Mitra et al., 2017). In addition, in most cases of atherosclerosis, plaques appear in the carotid bifurcation and aortic arch, which are regions with DF (Gimbrone and Garcia-Cardeña, 2013), thereby suggesting a relationship between hemodynamics and atherosclerosis progression. Cav-1 expression is reduced in DF as compared to UF, which indicates that Cav-1 regulation depends on the flow (Harding et al., 2018). Furthermore, as described by Harding et al. (2018), expression of active eNOS phosphorylated at Ser^1177^ is 50% lower in DF aortic arch than in UF abdominal aorta.

In this way, caveolae, Cav-1, and glycocalyx play an important role in vascular homeostasis, contributing to adequate NO production. However, atherosclerosis and hypertension impair NO bioavailability due to lower eNOS expression, eNOS inactivation, changes in Cav-1 expression or NOX4 activity, and eNOS uncoupling, leading to deleterious ROS overproduction.

## Discussion

In this review, we discuss recent findings about the physiological role of glycocalyx and caveolae, to maintenance of vascular tone, as well as alterations in these structures that are associated with the development of atherosclerosis and hypertension.

Even the glycocalyx has been reported as the primary sensor to mechano-transduction, a study demonstrated that caveolae show a unique molecular topography (Schnitzer et al., 1995) and may act as either mechano-sensors or transducers (Uittenbogaard et al., 2000; Gratton et al., 2004). Therefore, it could exist a relationship between glycocalyx and caveolae that is sensitive to feel mechanical forces and start the mechano-transduction process, and to promote an effective control of vascular tone.

During the first 30 min of exposition to shear stress, aortic and vein ECs (BAEC, bovine aortic EC; HUVEC, human umbilical vein EC) presented an accumulation of heparan sulfate and glypican-1 in the cell junctions. In contrast, there were no movement from chondroitin sulfate, syndecan-1, and Cav-1, indicating that these components and particularly the caveolae structure are anchored sufficiently to resist against initial exposure to shear stress (Zeng et al., 2013). On the other hand, the chronic shear stress (6 h) stimulated by changes in flow intensity in perfused lung microvessels was able to increase fivefold Cav-1 expression and sixfold caveolae density at the luminal surface compared with no-flow control, which contributed to enhanced mechano-sensivity in cultured ECs (Rizzo et al., 2003). Moreover, the glypican-1 inhibition, but not syndecan-1, blocked eNOS activation induced by shear stress in mammalian epithelial cells (Ebong et al., 2014). These studies clarify the activation of glypican-1-caveolae-eNOS-NO pathway under mechanical stimulus. In this way, there is a relationship between glycocalyx and caveolae, where they are exchanging information all the time, and both are susceptible to reorganization underlie different stimulus to regulate vascular tone and promote vascular homeostasis.

Furthermore, the relationship between glycocalyx and caveolae is not only observed during shear stress and mechano-transduction. Catestatin is a peptide derived from glycoprotein chromogranin A, which is expressed in neuroendocrine and cardiac cells. Catestatin acts in several organs/systems, including the cardiovascular system. The catestatin was applied to BAECs, where it co-localizes with heparan sulfate proteoglycans, promoting endocytosis of caveolae and inducing Cav-1 internalization, followed by eNOS phosphorylation at Serine^1179^ (Fornero et al., 2014). Therefore, the glycocalyx and caveolae collaborate with each other during the catestatin-dependent eNOS-activation.

Glycocalyx contributes to maintaining vascular homeostasis, and it protects the EC surface. Thus, its disruption and shedding contribute to the development of cardiovascular diseases. Therefore, preventing its degradation is important. In a review, Becker et al. (2010) brought together the pharmacological options to avoid glycocalyx shedding and perturbation, which included hydrocortisone application, use of antithrombin III, and infusion of human plasma albumin, which seems to be the effective treatment. Apart from that, rat fat pad ECs supplemented with heparan sulfate or sphingosine 1-phosphate regenerate glycocalyx (Mensah et al., 2017). Another agent, sulodexide, which is a mixture of heparan sulfate and dermatan sulfate (Coccheri and Mannello, 2013), also reconstitutes glycocalyx in patients (Broekhuizen et al., 2010). Nuclear magnetic resonance analysis demonstrated that Krüppel-like Factor 2 (KLF2) inhibits endothelial glycolysis and contributes to hexosamine and glucuronic acid biosynthesis (Wang et al., 2020). In addition, inflammatory cytokines like TNF-α, interleukin-1β, interleukin-6, and interleukin-8, as well as ROS can activate heparanase, MMPs, and hyaluronidase, which are enzymes that cleave chains of glycocalyx constituents (Uchimido et al., 2019). Hence, antioxidant drugs and direct inhibition of cytokines may be another option to prevent glycocalyx degradation.

In the last 20 years, many studies have evidenced the relevance of caveolins by using Cav-1 knockout mice with cardiovascular abnormalities (Li et al., 2005; Lian et al., 2019). As suggested by Forrester et al. (2017), Cav-1 may be the therapeutic target to treat hypertension and atherosclerosis. However, the role of Cav-1 is controversial in the literature. It has dual action: Cav-1 impairs vascular functions in specific cases and at the same time, it seems to be essential to maintain vascular homeostasis. Hypertension induced by AngII in Cav-1 knockout mice does not develop vascular remodeling, which means that Cav-1 deletion attenuates vascular hypertrophy and perivascular fibrosis (Forrester et al., 2017). On the other hand, on the basis of the mouse hypoxia model, reduced Cav-1 expression increases ROS production, and macrophages isolated from Cav-1 knockout mice and Cav-1 knockdown siRNA in human lung fibroblasts enhances ROS production. The absence of Cav-1 negatively regulates NOX-mediated ROS production (Chen et al., 2014). Additionally, Cav1-deficient mice exhibit pulmonary hypertension, impairment of left ventricular diastolic function, increased pulmonary vascular remodeling, and right ventricle hypertrophy and decreased contractility (Zhao et al., 2002). Furthermore, the lack of Cav-1 improves NO-dependent vascular function and produces higher levels of NO (Drab et al., 2001; Razani et al., 2001; Murata et al., 2007), which suggests that Cav-1 impairs vascular function and contributes to the development of cardiovascular diseases. Notwithstanding, various studies have shown that the presence of Cav-1 is mandatory for eNOS activation (Chen et al., 2012).

Navarro et al. (2014) suggested gene or cell therapy as antisense and siRNA approaches to target Cav-1 directly or to modulate caveolar and lipid levels as an alternative intervention either to increase or to decrease Cav-1 expression. Moreover, activation of some GPCRs would allow to control or to re-program Cav-1 expression levels to explore therapeutic outcomes in cardiovascular diseases. Besides, the pathway glypican-1/caveolin-1/eNOS/NO should be better explored for better understanding of this path and possible therapeutic treatments.

## Conclusion

**FIGURE 1:**
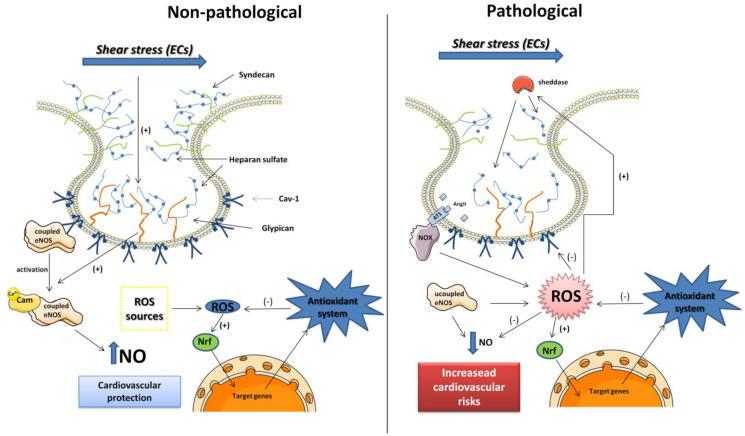
Schematic representation of caveola and glycocalyx proteins in endothelial cells (ECs) under non-pathological and pathological conditions. In non-pathological conditions, the glycocalyx proteins are intact and the coupled eNOS (dimeric form) is inactivated linked to Cav-1 protein. The eNOS is activated by shear stress mediated by Glypican-1 mechano-transduction as well as increase in calcium levels. The NO bioavailability can protect the cardiovascular system, and ROS produced by different sources stimulates the antioxidant system that also protects the cells of oxidative damage. In pathological conditions, the rise in ROS production mediated by pro-oxidant molecules as Ang II overtakes the antioxidant defenses. The ROS can degrade Cav-1, the eNOS is uncoupled (monomeric) that can be source of ROS, and NO bioavailability is reduced. ROS also stimulates the sheddases activity clivating the heparan sulfate from glypican and syndecan losing its ability to induce eNOS activation mediated by shear stress. Those frames with low NO levels can potentiate the cardiovascular risks.

The structure and function of both glycocalyx and caveolae are essential for maintenance of vascular homeostasis. Under pathological conditions, that are associated with ROS synthesis and release, the glycocalyx and caveolae structure and function could change, leading to impairment of their physiological function, which are the hallmark of cardiovascular diseases (see Figure 1).

## Author Contributions

SRP and LMB conceived the original scope of this manuscript. SRP, TP, MP, and LMB wrote specific sections. TP made the schematic representation of Figure 1. SRP and LMB critically reviewed and revised the final manuscript. All the authors have equally made a substantial intellectual contribution to this work in organizing and writing the manuscript, and all of them have approved it for submission.

## Conflict of Interest

The authors declare that the research was conducted in the absence of any commercial or financial relationships that could be construed as a potential conflict of interest.
